# The highest-ranking rooster has priority to announce the break of dawn

**DOI:** 10.1038/srep11683

**Published:** 2015-07-23

**Authors:** Tsuyoshi Shimmura, Shosei Ohashi, Takashi Yoshimura

**Affiliations:** 1 Laboratory of Animal Physiology; 2 Avian Bioscience Research Center, Graduate School of Bioagricultural Sciences; 3 Institute of Transformative Bio-molecules (WPI-ITbM), Nagoya University, Nagoya, Aichi 464-8601; 4 Division of Seasonal Biology, National Institute for Basic Biology, Okazaki, Aichi 444-8585; 5 Department of Basic Biology, SOKENDAI (The Graduate University for Advanced Studies), Miura, Kanagawa 240-0193, Japan

## Abstract

The “cock-a-doodle-doo” crowing of roosters, which symbolizes the break of dawn in many cultures, is controlled by the circadian clock. When one rooster announces the break of dawn, others in the vicinity immediately follow. Chickens are highly social animals, and they develop a linear and fixed hierarchy in small groups. We found that when chickens were housed in small groups, the top-ranking rooster determined the timing of predawn crowing. Specifically, the top-ranking rooster always started to crow first, followed by its subordinates, in descending order of social rank. When the top-ranking rooster was physically removed from a group, the second-ranking rooster initiated crowing. The presence of a dominant rooster significantly reduced the number of predawn crows in subordinates. However, the number of crows induced by external stimuli was independent of social rank, confirming that subordinates have the ability to crow. Although the timing of subordinates’ predawn crowing was strongly dependent on that of the top-ranking rooster, free-running periods of body temperature rhythms differed among individuals, and crowing rhythm did not entrain to a crowing sound stimulus. These results indicate that in a group situation, the top-ranking rooster has priority to announce the break of dawn, and that subordinate roosters are patient enough to wait for the top-ranking rooster’s first crow every morning and thus compromise their circadian clock for social reasons.

The crowing of roosters has informed human beings of the coming of morning since the Indus civilization (B.C. 2600–1800)^1^,^2^, and this sound symbolizes the break of dawn in many cultures. Previously, we showed that the timing of roosters’ predawn crowing is regulated by the circadian clock, an internal biological clock with a period about 24 hours^3^. Although external stimuli such as light and crowing by other individuals also induce crowing, the magnitude of this induction is also controlled by the circadian clock^3^. Entrainment is synchronization of a circadian clock to environmental cycles. The ambient light-dark cycle serves as the most effective synchronizer for the circadian clock. However, once organisms are transferred to constant conditions (such as constant darkness), free-running rhythms can be observed. Interestingly, in some species, entrainment of circadian clock to social cues is observed in the absence of other time cues^4^. For example, fruit flies exhibit more coherent group rhythms in constant darkness when housed together than when housed individually^5^. This group synchronization is disrupted by the introduction of arrhythmic flies into the group. Such synchronization may facilitate tight group organization, effective hunting and foraging, and reproduction^6^.

When one rooster breaks the dawn, others in the neighborhood have a higher probability of crowing^7^. Chickens are highly social animals^8^, and crowing is thought to be a means of advertising their territories^9^, thus avoiding the risk of direct aggressive interactions^10^. When the group size is small enough for each bird to recognize the others (generally, less than 10 individuals), chickens develop a linear and fixed hierarchy, also known as the “pecking order”^11^,^12^. In such groups, the behaviors of each rooster reflect the social hierarchy, and higher-ranking roosters have priority for food, mating, and resources in the pen such as nests and roosting places^8^,^13^. Thus, the pecking order forms the basis of social behavior in chickens. Here, we show that the top-ranking rooster also has priority to determine the timing of predawn crowing, and that subordinates are obedient to the top-ranking rooster in a group situation.

## Results

### The top-ranking rooster announces the break of dawn

**Figure 1:**
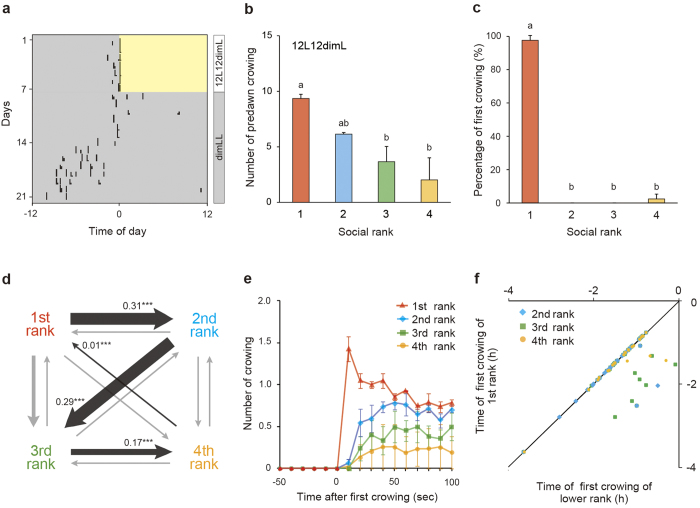
The top-ranking rooster in a group announces the break of dawn. (**a**) Representative actogram of crowing behavior under 12 hour light:12 hour dim light (12L12dimL) and constant dim light (dimLL) conditions for one individual from one of three rooster groups. The light and dim light periods are indicated by yellow and gray backgrounds, respectively. (**b**) Higher-ranking roosters tended to crow more frequently than lower-ranking roosters under the 12L12dimL condition (*F*_3,8_ = 6.8, *P* < 0.05, ANOVA, Tukey-Kramer’s test; mean + SEM, n = 3 groups). Different characters indicate significant differences. The data follows the normal distribution (χ^2^ = 3.8, *P* > 0.05). (**c**) The first-ranking rooster started to crow first every morning (*F*_3,8 = _124.0, *P* < 0.01, ANOVA, Tukey-Kramer’s test; mean + SEM, n = 3 groups). The data were arc-sin transformed before analysis and back-transformed data was shown in the figure. (**d**) Transition diagram of crowing order between social ranks showed that roosters start to crow in descending order of social rank. The proportion of the transition in relation to the whole is indicated by line weight. Significantly increased transitions are shown by black lines, with their proportion and significance (^***^P < 0.001), and the other transitions are shown by light gray lines. (**e**) Lower-ranking roosters immediately followed the first-ranking rooster’s predawn crowing (mean ± SEM, n = 3 groups). (**f**) A strong positive correlation was observed between the timing of first crowing of the top-ranking rooster and those of its subordinates (1st and 2nd rank: *R* = 0.98, *P* < 0.01; 1st and 3rd rank: *R* = 0.85, *P* < 0.01; 1st and 4th rank: *R* = 0.78, *P* < 0.01, Pearson’s correlation). Time 0 indicates light-onset time.

Four inbred roosters of the PNP strain were kept in a group cage in order to fix and determine the dominance hierarchy. Roosters were then introduced in groups of four into individual experimental cages in a light- and sound-tight room (Supplementary Fig. S1a). A total of three groups were examined in each experiment. We analyzed the relationship between crowing behavior and social ranks under a 12-h light:12-h dim light (12L12dimL) condition for 14 days, and then under a constant dim light (dimLL) condition for 14 days. Consistent with the results of a previous study^3^, anticipatory predawn crowing was observed approximately 2 hours (1.83 ± 0.23 h) before light onset under the 12L12dimL condition (Fig. 1a). Simple linear and stable hierarchies were observed in all groups. Higher-ranking roosters tended to crow more than lower-ranking roosters under the 12L12dimL condition (Fig. 1b). The top-ranking rooster almost always started to crow first every morning (97.6 ± 2.9%; Fig. 1c), followed by lower-ranking roosters in descending order of social rank (Fig. 1d). When we looked at temporal changes in the incidence of predawn crowing in detail, subordinates followed the top-ranking rooster within a few tens of seconds (Fig. 1e). We also confirmed that lower-ranking roosters crowed less than higher-ranking roosters over this short time scale (i.e., ~100 s) (Fig. 1e). Even though the timing of first crowing by the top-ranking rooster varied each day (Fig. 1a), the timing of subordinates’ first crowing was strongly correlated with that of the top-ranking rooster (Fig. 1f). These results suggested that the timing of lower-ranking roosters’ crowing is dependent on that of the top-ranking rooster. Consistent results were also observed in free-running subjective predawn crowing under the dimLL condition (Supplementary Fig. S1b–f).

### Second-ranking rooster initiates crowing when the top-ranking rooster is removed

**Figure 2:**
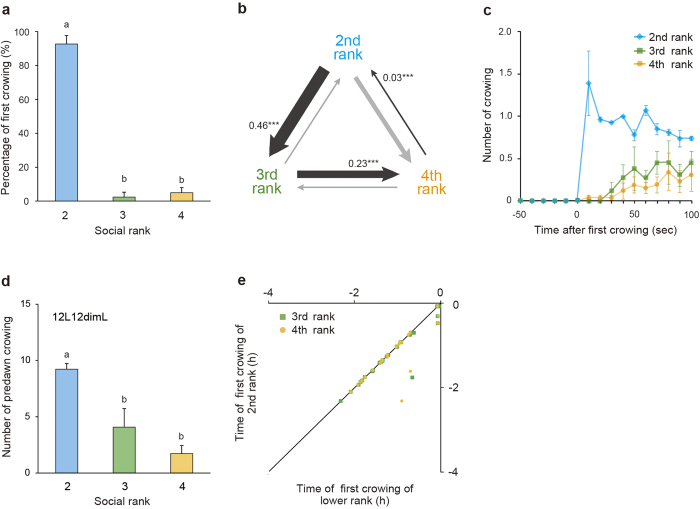
The second-ranking rooster initiates crowing when the first-ranking rooster is removed from the group. (**a**) The second-ranking rooster started to crow first in the group when the first-ranking rooster was physically removed. (*F*_2,6_ = 274.8, *P* < 0.01, ANOVA, Tukey-Kramer’s test; mean + SEM, n = 3 groups). Different characters indicate significant differences. The data were arc-sin transformed before analysis and back-transformed data was shown in the figure. (**b**) Transition diagram of crowing order between social ranks showing that roosters started to crow in descending order of social rank. The proportion of the transition in relation to the whole is indicated by line weight. The significantly increased transitions are indicated by black lines, with their proportion and significance (^***^P < 0.001), and the other transitions are indicated by light gray lines. (**c**) Lower-ranking roosters immediately followed the second-ranking rooster’s first crowing (mean ± SEM, n = 3 groups). (**d**) Higher-ranking roosters crowed more frequently than lower-ranking roosters under the 12L12dimL condition (*F*_2,6_ = 12.6, *P* < 0.01, ANOVA, Tukey-Kramer’s test; mean + SEM, n = 3 groups). The data follows the normal distribution (χ^2^ = 0.8, *P* > 0.05). (**e**) A strong positive correlation was observed between the timing of first crowing of the second ranking rooster and those of lower-ranking roosters (2nd and 3rd rank: *R* = 0.95, *P* < 0.01; 2nd and 4th rank: *R* = 0.85, *P* < 0.01, Pearson’s correlation). Time 0 indicates light-onset time.

We next examined the effect of physical removal of the top-ranking rooster on subordinates’ crowing behavior. When the top-ranking rooster was removed, the second ranking rooster started to crow first (92.7 ± 5.1%; Fig. 2a), and the third- and fourth-ranking roosters immediately followed the second-ranking rooster’s crow (Fig. 2b,c). In addition, lower-ranking roosters crowed less frequently (Fig. 2c,d). As in the case of the groups of four (Fig. 1f), the timing of first crowing of the second-ranking rooster and its subordinates were strongly correlated (Fig. 2e).

### Free-running periods of crowing rhythms coincide within a group

**Figure 3:**
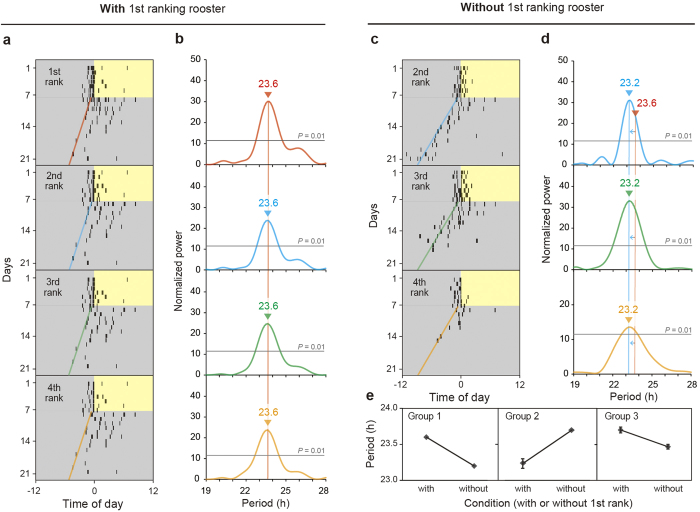
Free-running periods of individual crowing rhythms are identical within groups. (**a,c**) Representative actograms of crowing behavior of all individuals in one of 3 rooster groups under 12L12dimL and dimLL conditions in the presence (**a**) or absence (**c**) of the first-ranking rooster. (**b,d**) Periodogram analysis of crowing rhythms under the dimLL condition in the presence (**b**) or absence (**d**) of the first-ranking rooster. Free-running periods of crowing behaviors were identical within groups. However, the free-running periods were altered when the first-ranking rooster was physically removed from the group. (**e**) Although the first-ranking rooster always crowed first within a group, the free-running periods of crowing behavior were not always shorter in the presence of the first-ranking rooster than in its absence (mean ± SEM, n = 3 groups). Note that (**a)** is identical with Fig. 1A of Shimmura and Yoshimura (2013)^3^, because this study analyzed data from the same animals used in the previous study.

Because the timing of subordinates’ crowing was closely related to that of the top-ranking rooster, we predicted that the crowing rhythms of subordinates would coincide with that of the top-ranking rooster. Indeed, periodogram analysis of crowing rhythms demonstrated such coincidence within groups (Fig. 3a,b). In addition, when the top-ranking rooster was physically removed from groups, the free-running period of subordinates’ crowing was altered (Fig. 3c,d): after the removal of the top-ranking rooster, shortening of free-running rhythms was observed in two groups, and lengthening of the rhythm was observed in one group (Fig. 3e). Although the top-ranking rooster always crowed first every morning, these results suggested that the free-running period of the top-ranking rooster is not always the shortest within a group.

### All roosters have different free-running periods of body temperature rhythms

**Figure 4:**
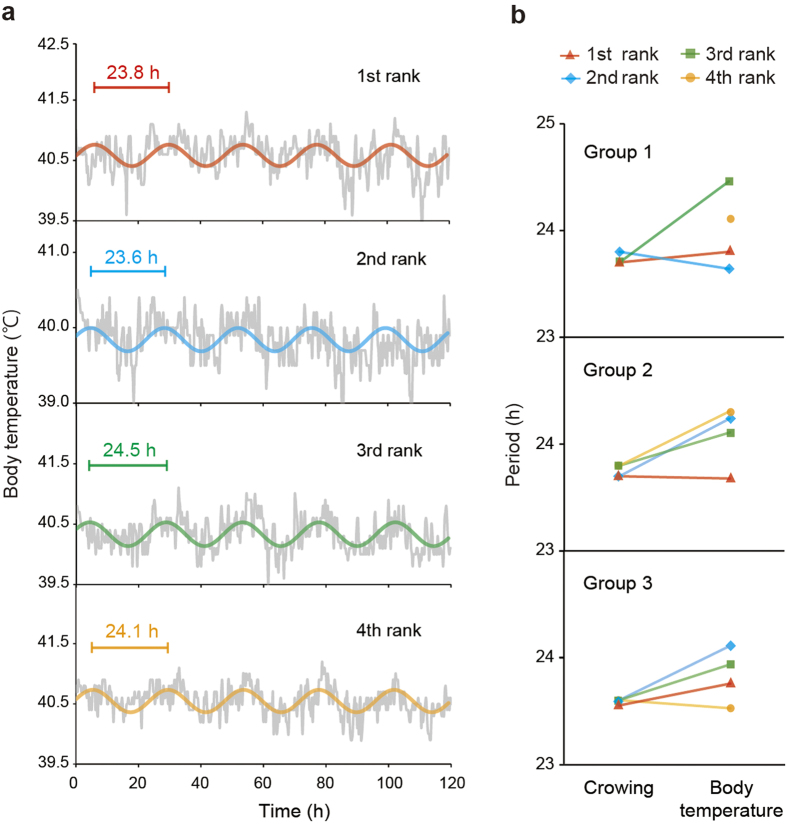
All roosters have different free-running periods of body temperature rhythms. (**a**) Representative free-running rhythms of body temperature of all individuals in one of 3 rooster groups, fitted with cosine curves (1st rank: *R* = 0.47, *P* < 0.01; 2nd rank: *R* = 0.35, *P* < 0.01; 3rd rank: *R* = 0.50, *P* < 0.01; 4th rank: *R* = 0.58, *P* < 0.01, Pearson’s correlation). (**b**) Although the free-running periods of crowing rhythms were identical within groups, those of body temperature rhythms differed among individuals. Note that the 4th-ranking rooster stopped crowing under dimLL condition in group 1.

We next examined whether the circadian clocks of subordinates are entrained to the top ranking rooster’s circadian clock. It is well established that the locomotor activity rhythms of galliform birds (i.e., chicken and quail) are not clear^14^. To address this issue, we examined roosters’ body temperature rhythms under dimLL conditions and fitted them to cosine curves. This analysis revealed that the free-running periods of body temperature rhythms differed among individuals kept within the same group (Fig. 4a,b), even though the free-running periods of crowing rhythms appeared to be the same within groups (Fig. 3).

### Anticipatory predawn crowing rhythm does not entrain to a timed crowing sound stimulus

**Figure 5:**
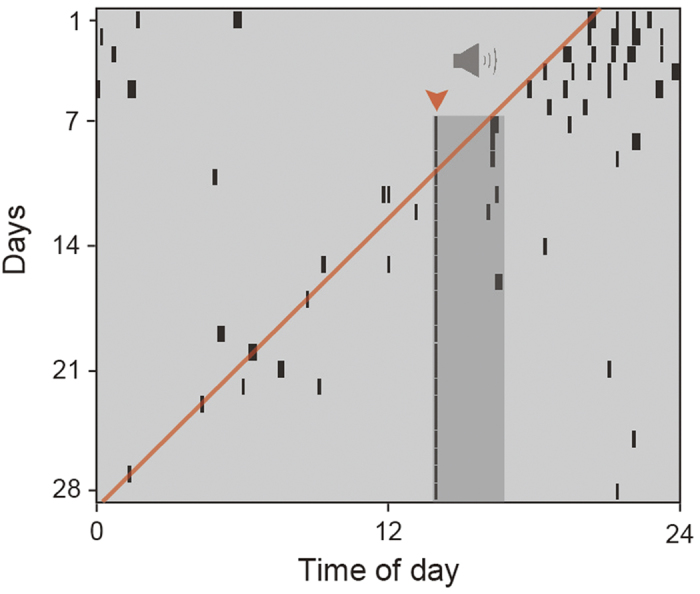
Predawn anticipatory crowing does not entrain to a timed crowing sound stimulus under the dimLL condition. Crowing sound stimulus of familiar roosters was given for 3 hours each day (dark gray shade). Although sound stimulus–induced crowing was observed immediately after the onset of the sound stimulus (arrowhead), anticipatory predawn crowing continued to free-run under the dimLL condition. Data from 1 individual from one of 3 rooster groups.

Finally, we investigated whether free-running crowing rhythms can entrain to the crowing sound of other individuals under dimLL conditions. Although we observed induction of crowing by sound stimulus, anticipatory predawn crowing did not entrain to the timed sound stimulus (Fig. 5).

## Discussion

In this study, we discovered that the highest-ranking rooster crows first every morning, followed by its subordinates in descending order of their social rank (Fig. 1c–e). Although anticipatory predawn crowing was usually observed approximately 2 hours before light onset, the timing of the top-ranking rooster’s first predawn crow varied to some extent (Fig. 1a). Crowing order, which reflects social rank, is strictly conserved even when the timing of the top-ranking rooster’s first crow is advanced or delayed each day (Fig. 1f). We also observed that lower-ranking roosters crowed less than higher-ranking roosters (Fig. 1b,e). Previous studies reported that the presence of a dominant rooster suppresses subordinates’ crowing^15^,^16^. By contrast, when we examined the effect of external stimuli (light stimulus or crowing sound stimulus) on crowing behavior, the percentage of first crowing (Supplementary Fig. S2a,b) and the number of crows (Supplementary Fig. S2c,d) were independent of social rank. These results suggested that although lower-ranking roosters do have the potential to crow, predawn crowing by subordinates is repressed by the presence of dominant roosters. This idea is also supported by the experiment in which we removed the top-ranking rooster. In the presence of the top-ranking rooster, the crowing of second ranking rooster was suppressed (Fig. 1c–e). However, once the top-ranking rooster is physically removed from the group, the second-ranking rooster behaves as if he is the top-ranking rooster (Fig. 2).

Because the timing of subordinates’ crowing was closely related with that of the top-ranking rooster, it was reasonable to speculate that the crowing rhythms of subordinates were entrained to the crowing rhythm of the top-ranking rooster. Indeed, the free-running periods of subordinates’ crowing were coincident with that of the top-ranking rooster under dimLL conditions (Fig. 3a,b). When the top-ranking rooster was removed from groups, the second-ranking rooster took its place, and the free-running periods of the remaining three individuals were altered (Fig. 3c,d). Notably, the lower-ranking rooster sometimes crowed first in this situation (Fig. 3c), probably because the second-ranking rooster was not as able as the top-ranking rooster to repress lower-ranking roosters (Supplementary Fig. S3). We then analyzed the free-running rhythms of body temperature rhythms, but found that they differed among individuals (Fig. 4). In addition, anticipatory predawn crowing did not entrain to the timed sound stimulus of other roosters’ crowing (Fig. 5). This result was in marked contrast with the circadian clock of songbirds, which can entrain to a sound stimulus^17^,^18^. All of these results suggested that the circadian clocks of subordinates are not entrained to that of the top-ranking rooster, and an identical free-running period observed within a group appeared to be a “masking response”, i.e., a direct response to environmental stimuli.

Because the top-ranking rooster always started to crow first each morning (Fig. 1, 2, Supplementary Fig. S1), it was also reasonable to speculate that the free-running period of the top-ranking rooster is shorter than those of its subordinates, or that the rooster whose free-running period is the shortest would become the highest-ranking rooster. However, both of these hypotheses are unlikely to be true, because the top-ranking rooster did not always have the shortest free-running period (Fig. 3e, 4). However, interestingly, the top-ranking rooster showed similar free-running period in both crowing and body temperature (Fig. 4b), which suggest that top-ranking rooster start crowing on his own timing. On the other hands, although the subordinate rooters also have their own free-running rhythm of body temperature, they appear to keep a pace with the top-ranking rooster. Taken together, the data suggest that subordinate roosters have the potential to crow, but they are patient enough to wait for the top-ranking rooster’s first crow.

In summary, in this study, we demonstrated that the highest-ranking rooster has priority to announce the break of dawn, based on his own circadian clock, and that subordinate roosters compromise their clocks for social reason and wait for the top-ranking roosters’ first crow every morning.

## Methods

### Animals and experimental environment

Inbred roosters of the PNP strain were used in this study^19^. Each group consisted of four roosters. These four roosters were kept in four individual cages (44 cm × 39 cm × 74 cm) within a single light- and sound-tight room (Supplementary Fig. S1a). A total of three groups were examined in each experiment using the same four cages and room. Fully matured 30-week-old roosters that exhibited crowing behavior were used for experiments. The room temperature was set at 20.0 °C. The roosters had ad libitum access to water and feed. Animals were treated in accordance with the guidelines of Nagoya University. All experimental protocols were approved by Nagoya University.

### Dominance hierarchy

Four roosters were initially kept in a group cage in order to fix and determine the dominance hierarchy. For focal behavior observation, all roosters were individually marked using colored leg rings. Direct visual observation of aggression was conducted in the daytime. Aggressive behaviors (aggressive pecking, displacing, chasing, and threatening) were recorded, and both winner and loser were noted^20^. From the record of the winners and losers of these aggressive interactions, we calculated the dominance value of individual roosters by using the index of Clutton-Brock (ICB)^21^,^22^, calculated using the following formula:

Dominant value 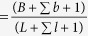


where *B* = number of roosters that an individual beat; Σ*b* = total number that they beat excluding the subject; *L* = number of roosters that the individual lost to; Σ*l* = total number that they lost to excluding the subject. This index takes into account the success of opponents, so that an individual’s score is determined by the score of the individuals it dominated and of those that dominated that individual. The formula is especially effective in the case of a linear and fixed hierarchy, such as that of domestic chickens^23^. We also calculated the linearity in each group, using Landau’s index of linearity^24^. Normalized index values (h) range from 0 (nonlinear) to 1 (perfectly linear), and h ≥ 0.9 would be a reasonable (although arbitrary) cutoff criterion for ‘strong’, nearly linear hierarchies. In this study, the mean (± SEM) index value of linearity (h) was 0.97 ± 0.02, confirming that the hierarchies were nearly linear. Therefore, we used the dominance values to identify the rank of each rooster: 7.4 ± 0.6 (1st rank), 2.2 ± 0.2 (2nd rank), 0.6 ± 0.1 (3rd rank), and 0.2 ± 0.0 (4th rank). The dominance values were differed significantly between ranks (*F*_3,8_ = 137.8, *P* < 0.01, ANOVA, Tukey-Kramer’s test). Consistent with previous reports^11^,^12^,^25^, a simple linear hierarchy was observed in this study. After each experiment, we introduced the four roosters back into a group cage and confirmed that the social rank remained the same throughout the experiment by examining the aggressive interactions as described above^20^.

### Behavioral observation

Roosters were then introduced into individual experimental cages to record individual crowing behavior (Supplementary Fig. S1a). This also helped to avoid reduction in the frequency of crowing by lower-ranking roosters due to aggressive pecking. All roosters were kept under 12L12dimL condition for 1 week to allow adaptation before the beginning of each experiment. The lighting was adjusted to give an intensity of 100 lux for regular light and <0.1 lux for dim light at the height of the roosters’ heads. The crowing of roosters was recorded all day using an IC recorder (ICD-UX300F, Sony) and an HD recorder (DMR-XP200, Panasonic) connected to a digital video camera (HDR-XR550V, Sony) equipped with a near-infrared illuminator (K-Light, Keiyo Techno). Because roosters exhibit elevation and extension of the head prior to and during crowing^26^, the crowing of each individual rooster could be easily distinguished from the sound and video recordings.

### Sound and light stimuli

When we investigated the entrainment to the crowing sound (Fig. 5), 100 dB sound stimuli were given for 3 hours. Using Sound Forge Audio Studio v9.0 (Sony), the sound stimuli of a familiar rooster’ crowing were edited as follows: six different crows/min (one crowing/10 sec) × 180 replicates (=3-h sound stimuli). The sound stimuli were presented using a speaker (SRS-T10PC, Sony) placed in front of the cage. To investigate the effect of light stimuli on the order of crowing (Supplementary Fig. S2a,c), we counted the number of crows during a 30-min exposure to a 100 Lux light stimulus at light-onset^3^. For the effect of sound stimuli (Supplementary Fig. S2b, d), 100 dB sound stimuli were given for 30 min^3^.

### Body temperature recording

Body temperature was measured at 15 min interval using a temperature data logger (Thermochron iButtons, Maxim Integrated Products; temperature accuracy: ±0.5 °C), implanted subcutaneously on the neck under anesthesia.

## Statistical analysis

Before the statistical tests mentioned below, we confirmed the normal distribution by test for difference of mean^27^. When normality could not be confirmed, the data were arc-sin transformed for proportion data and square-root transformed for count data^28^. One-way ANOVA was used to evaluate the effect of social rank. Significances of differences were evaluated by multiple comparisons using Tukey-Kramer’s test. The back-transformed data are shown in the figures. Behavioral sequences of crowing between ranks were analyzed calculating z-scores and testing significance^29^,^30^. Correlations between the timing of first crowing and social ranks were determined using Pearson’s correlation after time values were transformed into serial values (e.g. 0:00, 12:00, and 24:00 were transformed into 0, 0.5, and 1.0, respectively). Free-running periods of crowing under dimLL conditions were calculated by Lomb–Scargle periodogram analysis using the ClockLab software v2.61 (Actimetrics)^31^. Free-running periods of body temperature were calculated by cosine curve fitting using the Prism software v4.03 (GraphPad Software). The number of crowing was counted for 100 sec after first crow.

## Additional Information

**How to cite this article**: Shimmura, T. *et al.* The highest-ranking rooster has priority to announce the break of dawn. *Sci. Rep.* **5**, 11683; doi: 10.1038/srep11683 (2015).

## Supplementary Material

Supplementary Information
