# Supplementary material for: The highest-ranking rooster has priority to announce the break of dawn
Source: Sci Rep. 2015 Jul 23;5:11683. doi: 10.1038/srep11683 (PMC4512148; doi:10.1038/srep11683)
Supplement: Supplementary Information [file srep11683-s1.pdf]

Supplemental Information

The highest-ranking rooster has priority to announce the break of dawn

Tsuyoshi Shimmura, Shosei Ohashi, and Takashi Yoshimura

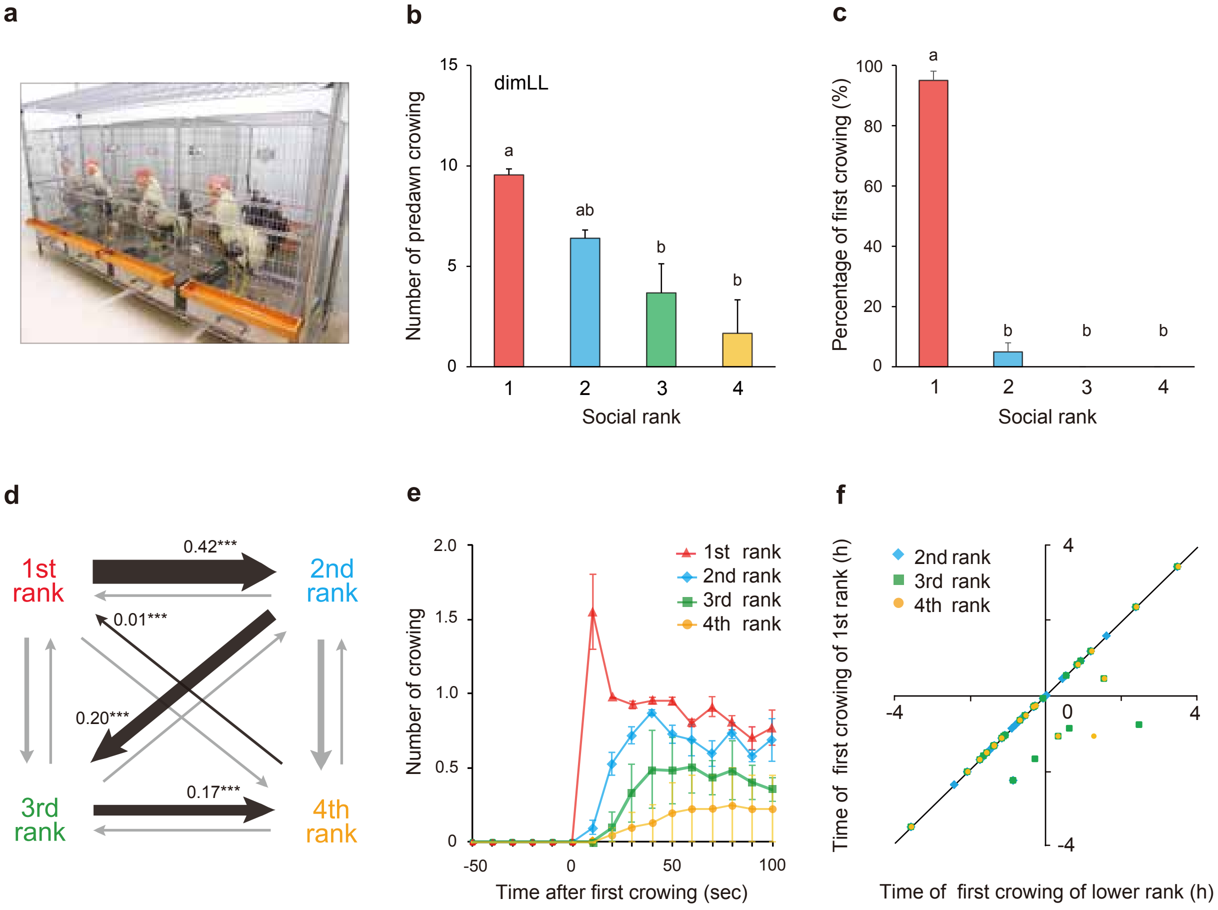

Supplementary Figure S1 | Top-ranking rooster announces the break of dawn even under dimLL conditions. (**a**) Recording of crowing behavior was performed in individual cages. (**b**) Higher-ranking roosters tended to crow more frequently than lower-ranking roosters, even under the dimLL condition ($F_{3,8}$ = 9.1, P < 0.01, ANOVA, Tukey-Kramer's test; means + SEM, n = 3 groups). Different characters indicate significant differences. The data follows the normal distribution (χ^2 = 2.3, P > 0.05). (**c**) First-ranking rooster started to crow first every morning, even under the dimLL condition ($F_{3,8}$ = 97.1, P < 0.01, ANOVA, Tukey-Kramer's test; means + SEM, n = 3 groups). The data were arc-sin transformed before analysis and back-transformed data was shown in the figure. (**d**) Transition diagram of crowing order between social ranks showed that roosters start to crow in descending order of social rank. The proportion of each transition in relation to the whole is indicated by line weight. Significantly increased transitions are indicated by black lines, with their proportion and significance ($^{***}P$ < 0.001), and the other transitions are indicated by light gray lines. (**e**) Lower-ranking roosters immediately followed the first-ranking rooster's crowing (mean ± SEM, n = 3 groups). (**f**) A strong positive correlation was observed between the timing of first crowing of the first-ranking rooster and those of its subordinates (1st and 2nd rank: R = 0.99, P < 0.01; 1st and 3rd rank: R = 0.94, P < 0.01; 1st and 4th rank: R = 0.94, P < 0.01, Pearson's correlation). Time 0 indicates the light-onset time of the previous 12L12dimL condition.

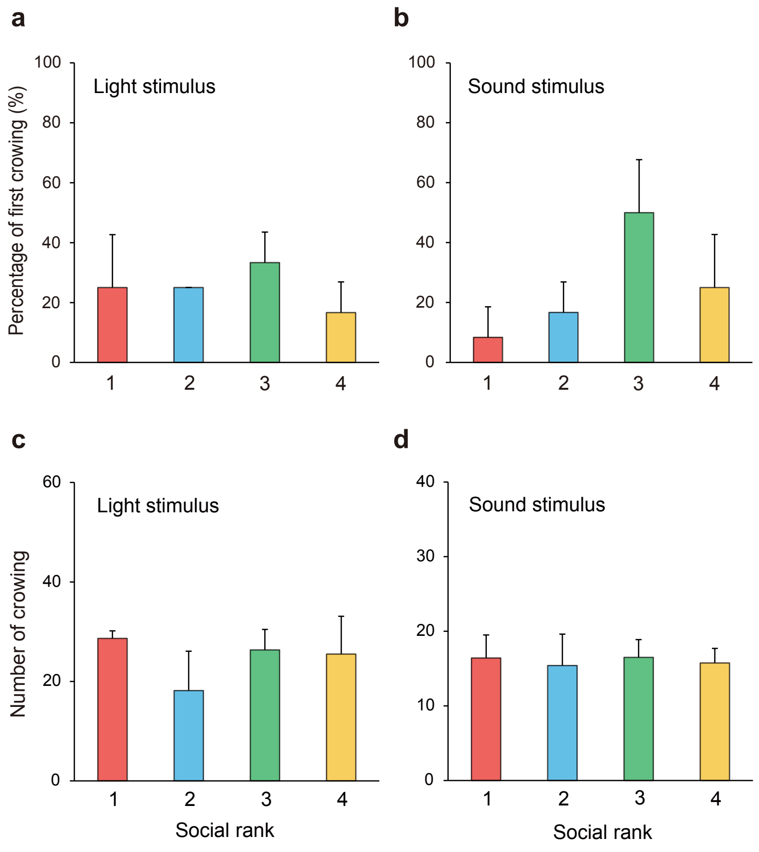

Supplementary Figure S2 | External stimulus–induced crowing is not related to the social rank. (**a, b**) Percentage of first crowing induced by light stimulus (**a**, $F_{3,8}$ = 0.5, P > 0.05, ANOVA, means + SEM, n = 3 groups) and crowing sound stimulus (**b**, $F_{3,8}$ = 2.4, P > 0.05, ANOVA, means + SEM, n = 3 groups) was not related to social rank. The data were arc-sin transformed before analysis and back-transformed data was shown in the figure. (**c, d**) In addition, the number of crowing induced by light stimulus (**c**, $F_{3,8}$ = 0.8, P > 0.05, ANOVA, means + SEM, n = 3 groups) and crowing sound stimulus (**d**, $F_{3,8}$ = 0.0, P > 0.05, ANOVA, means + SEM, n = 3 groups) was also not related to social rank. The data was arc-sine transformed before analysis and back-transformed data was shown in the figure.

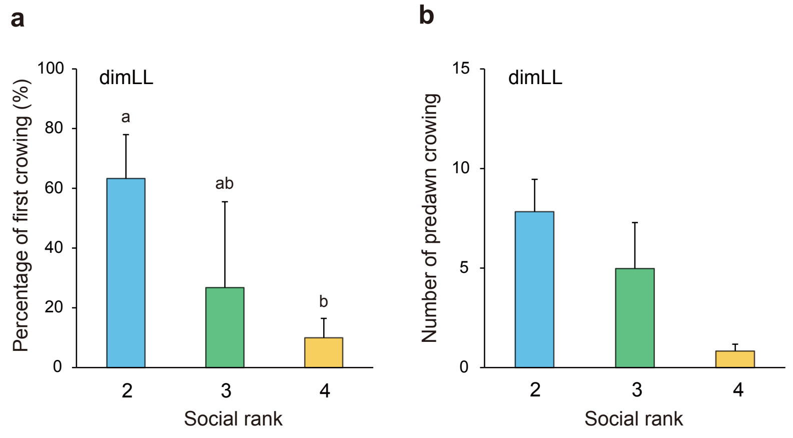

Supplementary Figure S3 | Repressive effects of the second-ranking rooster on lower-ranking roosters were not as strong as those of the first-ranking rooster under dimLL conditions. (See Supplemental Figure S1b,c) (**a**) Although the second-ranking rooster started to crow first ($F_{2,6}$ = 5,2, P < 0.05, ANOVA, Tukey-Kramer' s test; means + SEM, n = 3 groups), lower-ranking roosters also crowed first on some mornings. Different characters indicate significant differences. The data follows the normal distribution (χ^2 = 0.8, P > 0.05). (**b**) No significant difference in number of crowing was detected between ranks ($F_{2,6}$ = 4.6, P > 0.05, ANOVA, Tukey-Kramer' s test, means + SEM, n = 3 groups). The data follows the normal distribution (χ^2 = 0.4, P > 0.05).
